# Digital Voice Analysis as a Biomarker of Acromegaly

**DOI:** 10.1210/clinem/dgae689

**Published:** 2024-10-04

**Authors:** Konstantina Vouzouneraki, Fredrik Nylén, Jenny Holmberg, Tommy Olsson, Katarina Berinder, Charlotte Höybye, Maria Petersson, Sophie Bensing, Anna-Karin Åkerman, Henrik Borg, Bertil Ekman, Jonas Robért, Britt Edén Engström, Oskar Ragnarsson, Pia Burman, Per Dahlqvist

**Affiliations:** Department of Public Health and Clinical Medicine, Umeå University, SE-901 87 Umeå, Sweden; Department of Clinical Sciences, Umeå University, SE-901 87 Umeå, Sweden; Department of Clinical Sciences, Umeå University, SE-901 87 Umeå, Sweden; Department of Public Health and Clinical Medicine, Umeå University, SE-901 87 Umeå, Sweden; Department of Endocrinology, Karolinska University Hospital and Department of Molecular Medicine and Surgery, Karolinska Institutet, SE-171 76 Stockholm, Sweden; Department of Endocrinology, Karolinska University Hospital and Department of Molecular Medicine and Surgery, Karolinska Institutet, SE-171 76 Stockholm, Sweden; Department of Endocrinology, Karolinska University Hospital and Department of Molecular Medicine and Surgery, Karolinska Institutet, SE-171 76 Stockholm, Sweden; Department of Endocrinology, Karolinska University Hospital and Department of Molecular Medicine and Surgery, Karolinska Institutet, SE-171 76 Stockholm, Sweden; Department of Internal Medicine, Örebro University Hospital and Faculty of Health and Medical Sciences, Örebro University, SE-171 76 Örebro, Sweden; Department of Endocrinology, Skåne University Hospital, Lund University, SE-205 02 Malmö, Sweden; Department of Endocrinology and Department of Health, Medicine and Caring Sciences, Linköping University, SE-581 85 Linköping, Sweden; Department of Endocrinology and Department of Health, Medicine and Caring Sciences, Linköping University, SE-601 81 Norrköping, Sweden; Department of Endocrinology and Department of Health, Medicine and Caring Sciences, Linköping University, SE-581 85 Linköping, Sweden; Department of Medical Sciences, Endocrinology and Mineral Metabolism, Uppsala University, Uppsala University Hospital, SE-751 85 Uppsala, Sweden; Department of Endocrinology, Sahlgrenska University Hospital, SE-413 45 Gothenburg, Sweden; Department of Internal Medicine and Clinical Nutrition, Institute of Medicine at Sahlgrenska Academy, University of Gothenburg, SE-413 45 Gothenburg, Sweden; Wallenberg Centre for Molecular and Translational Medicine, Institute of Medicine, University of Gothenburg, SE-413 45 Gothenburg, Sweden; Department of Endocrinology, Skåne University Hospital, Lund University, SE-205 02 Malmö, Sweden; Department of Public Health and Clinical Medicine, Umeå University, SE-901 87 Umeå, Sweden

**Keywords:** acromegaly, digital voice analysis, machine learning, Voice Handicap Index

## Abstract

**Context:**

There is a considerable diagnostic delay in acromegaly, contributing to increased morbidity. Voice changes due to orofacial and laryngeal changes are common in acromegaly.

**Objective:**

Our aim was to explore the use of digital voice analysis as a biomarker for acromegaly using broad acoustic analysis and machine learning.

**Methods:**

Voice recordings from patients with acromegaly and matched controls were collected using a mobile phone at Swedish university hospitals. Anthropometric and clinical data and the Voice Handicap Index (VHI) were assessed. Digital voice analysis of a sustained and stable vowel [a] resulted in 3274 parameters, which were used for training of machine learning models classifying the speaker as “acromegaly” or “control.” The machine learning models were trained with 76% of the data and the remaining 24% was used to assess their performance. For comparison, voice recordings of 50 pairs of participants were assessed by 12 experienced endocrinologists.

**Results:**

We included 151 Swedish patients with acromegaly (13% biochemically active and 10% newly diagnosed) and 139 matched controls. The machine learning model identified patients with acromegaly more accurately (area under the receiver operating curve [ROC AUC] 0.84) than experienced endocrinologists (ROC AUC 0.69). Self-reported voice problems were more pronounced in patients with acromegaly than matched controls (median VHI 6 vs 2, *P* < .01) with higher prevalence of clinically significant voice handicap (VHI ≥20: 22.5% vs 3.6%).

**Conclusion:**

Digital voice analysis can identify patients with acromegaly from short voice recordings with high accuracy. Patients with acromegaly experience more voice disorders than matched controls.

One of the major challenges in acromegaly is its long diagnostic delay, which is partially due to the rarity of the disease but also the slow progress of nonspecific signs and symptoms (1). Once diagnosed, the long-standing disease has often already caused considerable suffering as a result of several partially irreversible complications associated with a pituitary tumor that is difficult to completely remove surgically (2–4). A frequent clinical observation in acromegaly is voice alteration, which could be a clue to diagnosis, and may also impair patient quality of life and work ability.

Acoustic analysis can be used to quantify voice and speech disturbances with increasing speed and power through modern digital processing techniques (5, 6). Previously described voice alterations in acromegaly include lower fundamental frequency of vocal fold cycles and higher perturbation markers than healthy controls, characterizing a hoarse deep voice (7–11). It has also been shown that patients have moderate self-perceived levels of voice impairment with a trend for alleviation after acromegaly treatment (10) as assessed using the Voice Handicap Index (VHI), an established clinical questionnaire for assessing experienced voice impairment (12, 13). The voice changes in acromegaly are likely due to the orofacial and laryngeal changes of long-standing growth hormone excess, including altered elasticity and increased volume of the vocal folds as well as skull deformities affecting the vocal tract (10, 14, 15). The observations of audible signs of disease onset have led to an increasing interest in voice as a digital biomarker for the identification and monitoring of several diseases with the aid of machine learning (5, 16, 17). However, previous acoustic studies of voice changes in individuals with acromegaly and controls have not explored the diagnostic potential of voice in acromegaly (7, 8). The vowel [a] has the highest prevalence (91%) across languages with known phonetic inventories (18) and, therefore, provides the best opportunity to offer a voice biomarker of diseases that can be transferred across languages.

The primary aim of this study was to develop a new diagnostic tool for acromegaly using digital voice analysis and machine learning algorithms to identify acromegalic voice characteristics and compare the model’s accuracy with voice assessment by experienced endocrinologists. A secondary aim was to evaluate self-reported voice symptoms in a large national cohort of patients with acromegaly.

## Materials and Methods

### Study Population

In this cross-sectional study, patients with acromegaly from the 7 university hospitals in Sweden were invited to participate between January 2021 and March 2023. The study included patients with both newly diagnosed and previously diagnosed acromegaly identified in the Swedish Pituitary Registry or in the local patient registries of each participating center. For each patient with acromegaly included, 1 control subject matched for gender and age (±5 years) was recruited from the same endocrine clinic’s staff or from patients with diagnoses not affecting voice or speech. Exclusion criteria were active smoking during the previous year, prior neck surgery or radiotherapy, known voice or speech problems from other causes, neurological conditions, medical treatment for asthma in the previous 4 weeks, upper respiratory tract infection in the previous week, and inability to read or understand the study material.

### Procedure

At the study visit, specialized research nurses made a standardized recording of the participant’s voice and data were collected, including date of diagnosis and treatments of acromegaly, duration of acromegalic symptoms, biochemical control and its duration assessed by the treating physician, size of pituitary adenoma at diagnosis, hormone levels (serum insulin-like growth factor-1 (IGF-1) and growth hormone levels ±6 months from the study visit and during the 5 years prior to the study), anthropometric data, and current medications. Physicians assessed biochemical control by evaluating current and previous IGF-1 and growth hormone levels; acromegaly-related symptoms, signs, and comorbidities; and the presence of any residual pituitary tumor. Laboratory analyses were made at accredited laboratories of each university hospital. All participants completed the VHI questionnaire. All participants gave their informed consent to participate in the study. The study was approved by the Swedish Ethics Review Board (registration number 2020-05417).

### Voice Recordings

Voice recordings were made in a quiet room by a research nurse using a mobile phone (Samsung Galaxy A40) configured for the study with a voice-recording application (Voice Recorder Pro, version 1.0.3), saving recordings as digital linear pulse-code modulation WAV files. During the recording, participants were asked to perform a sustained and stable vowel [a] and read a Swedish text of 160 words validated to assess patients with dysarthria (19). All produced sustained vowels [a] were manually identified and extracted for acoustic analysis. Further, read sentences were identified and extracted from the recordings.

### Voice Analysis

Similar to previous efforts to establish voice biomarkers of diseases (20), stationary sustained [a] was studied to support the identification of structural changes in the vocal tract without the interference of the dynamic changes in acoustic properties when speaking. A comprehensive analysis framework was constructed by consolidating 5 software frameworks for acoustic feature extraction: the Voice Analysis Toolbox (21), the Praat Voice Report (22), and openSMILE acoustic feature extractor's ComParE 2016, eGeMaps, and emobase feature sets (23, 24). The consolidated acoustic framework spanned 7450 acoustic analyses, which were applied to each sustained [a]. Features that could not be reliably extracted from all recordings (n = 957) were excluded. Of the 6493 features that could be extracted from all recordings, features with high correlation with at least 1 other feature (Pearson's r > 0.9) or 0 variance were not further considered in the analysis. The remaining 3274 acoustic features were used as predictors in subsequent modeling as described in “Machine Learning” below.

### Machine Learning

To build the voice classification model, 3 machine learning models (support vector machine [SVM], random forest [RF], and k-nearest neighbors [KNN]) were trained in a cross-validation procedure. The recordings of sustained [a] of all pairs of participants (patient with acromegaly, and age- and sex-matched control) with complete recordings were assigned to a training dataset and a test dataset by stratified random sampling, so that the proportion of patients in biochemical control occurred with a similar frequency in both datasets. As a result, 76% of samples were assigned to the training set in which the specific settings determining the properties and behavior (hyperparameters, see Table S1 (25)] of each machine learning model (SVM, RF, and KNN) were tuned using a 10-fold cross-validation procedure, in which the training data were divided into 10 parts (folds). The hyperparameters (combination of settings) of the models were then tuned iteratively in 9 folds (9/10th of the training data) with the goal of providing the best performance in predicting the data in the holdout (1/10th) fold, measured as the largest receiver operating characteristic area under the curve (ROC AUC). Hyperparameter search grids of 1000 values spaced according to a maximum entropy design were used in all tuning procedures. We employed a variogram range of 100 in the grid search for optimal hyperparameter settings. The search was repeated 10 times. In the final step of model training, the SVM, RF, and KNN models were combined into an ensemble by model stacking, with relative weights assigned by their relative strengths and weaknesses in predicting the training data.

The remaining (24%) pairs of participants were assigned to a test dataset that was not part of the model training procedures. The models were assessed based on the specificity, sensitivity, and ROC AUC of their performances in predicting acromegaly, assuming a .5 probability cutoff point.

### Endocrinologists' Assessment of Voice Recordings

Voice recordings of 50 pairs of patients with acromegaly and their matched controls were assessed by 12 experienced endocrinologists (co-authors: T.O., K.B., C.H., M.P., S.B., A.-K.Å., H.B., B.E., B.E.E., O.R., P.B., and P.D.) with expertise in pituitary diseases from all Swedish university hospitals using PsychoPy, an open source application for experiments in behavioral science (26). The participants in the experiment included the test set (31 pairs) and randomly chosen pairs from the training set (19 pairs). The experiment was performed on the listeners' own computers connected with identical headphones (AKG K271 MK II) with a good frequency response to ensure accurate playback of the recording. The experiment included extracted sound recordings of 1 sustained [a] and 4 short sentences from each participant, resulting in 500 voice stimuli. Additionally, 50 of the 500 voice stimuli were randomly chosen and duplicated to allow assessment of intrarater reliability. Thus, each endocrinologist assessed 550 voice samples, presented in random order, and registered a response of either “A” for “acromegaly” or “K” for “control” for each sample. The listener was allowed to repeat the stimulus at will, and unlimited times, before deciding. Voice samples in which the listener recognized the participant’s voice (as a colleague or patient of their own) were excluded.

### Voice Handicap Index

VHI is a validated self-rating questionnaire that quantifies the psychosocial consequences of a voice disorder. It includes 30 questions divided in 3 subdomains (functional, emotional, and physical), each with 10 questions and scores from 0 (never) to 4 (always), resulting in a maximum score of 40 in each subdomain and a maximum total score of 120 (13). Higher scores reflect more severe voice-related limitations (12). A total VHI score ≥20 indicates a significant level of experienced voice limitations (12). Thresholds for clinically meaningful differences in subdomain scores have been estimated to be in the range of 6 to 9 across studies of VHI implementations (12, 13, 27). A VHI score ≥10 in a VHI subdomain was employed here as a conservative threshold for indicating a perceived voice handicap.

### Statistics

Continuous variables are presented as median and interquartile range (IQR), and potential differences between cases and controls were tested using the Mann–Whitney U test. Categorical variables are presented as absolute numbers and percentages, and potential differences between subgroups were tested using the chi-square test. VHI scores were not normally distributed. The association between VHI scores and acromegaly was investigated by ordinal (proportional odds) regression, (univariate and both unadjusted and adjusted multivariable), with VHI score as dependent variable and acromegaly (yes/no [Y/N]) as independent variable and age, gender, body mass index (BMI), study site, and tertiary education (Y/N) as covariates. We also performed logistic regression in the acromegaly population (univariate and both unadjusted and adjusted multivariable), with total VHI ≥20 (Y/N) as independent variable and biochemical control (Y/N) as dependent variable with age, gender, BMI, and tertiary education (Y/N) as covariates. The assessment of endocrinologists' performance included the sensitivity and specificity of each listener predicting the speaker’s class (acromegaly Y/N). ROC AUCs and performance measure averages were also computed across all endocrinologists. The intrarater agreement for each listener was assessed using Cohen's kappa using the 50 duplicate sound samples. All calculations were performed using SPSS Statistics v29 for Mac (IBM), except for comparison of ROC AUCs, which utilized bootstrap analysis with pROC software (28). *P* < .05 was considered to be statistically significant.

## Results

### Patient Characteristics

We identified 301 Swedish patients with acromegaly of whom 99 declined participation and 51 were excluded in accordance with the exclusion criteria (ie, smoking/asthma [n = 20], language difficulties [n = 19], previous neck surgery [n = 9], neurological condition [n = 1]) or due to technical difficulties at voice recording (n = 2). Thus, the final analysis included 151 patients with acromegaly (39% women) with median (IQR) age at inclusion 57 (46-67) years and age at acromegaly diagnosis 44 (34-53) years (Table 1). Among these, 20 (13%) patients were newly diagnosed and not yet treated for acromegaly, and 15 (9.9%) patients were previously diagnosed and treated but considered not biochemically controlled by the treating endocrinologist. Among the 116 patients considered biochemically controlled by the treating endocrinologist, 11 patients had a registered IGF-1 at inclusion above the upper limit normal (ULN). Among these, 10 patients had IGF-1 ≤ 1.2 × ULN combined with low random growth hormone levels or several previous normal IGF-1 levels, whereas 1 patient had IGF-1 > 1.2 × ULN but multiple random growth hormone levels ≤0.4 µg/L and no clinical disease activity for 6 years.

**Table 1.** Baseline characteristics of patients with acromegaly

All patients with acromegaly (n = 151) (denominator for missing data)	
Age at inclusion, years	57 (46-67) (n = 151)
Female	59/151 (39%)
Male	92/151 (61%)
Age at acromegaly diagnosis, years	44 (34-53) (n = 150)
Time from acromegaly symptoms to diagnosis, years	5 (2-10) (n = 139)
Pituitary adenoma diameter at diagnosis, mm	15 (10-20) (n = 134)
Macroadenoma at diagnosis	105/135 (78%)
Serum IGF-1 ≤ULN	86/131 (66%)
Biochemical control by physician assessment	116/151 (77%)
Duration of biochemical control, years	9 (3-14) (n = 116]
**Treated patients with previously diagnosed acromegaly (n = 131)*^a^***	
Pituitary surgery	126/131 (96%)
Multiple surgeries	18/131 (14%)
Radiotherapy	39/129 (30%)
Current medical therapy	
Somatostatin analogue	39/131 (30%)
Pegvisomant	20/131 (15%)
Dopamine agonist	9/131 (7%)
Combination	12/131 (9%)
Pituitary hormone replacement	
Thyroid hormone	35/131 (27%)
Testosterone	29/131 (22%)
Cortisol	26/131 (20%)
Antidiuretic hormone	7/131 (5%)

Data are n (%) or median (IQR).

Abbreviations: IGF-1, insulin-like growth factor 1; IQR, interquartile range; ULN, upper limit of normal.

^*a*^Excludes 20 patients with newly diagnosed acromegaly who were still untreated.

To assess representability of the study cohort, a summary from the Swedish Pituitary Registry was made at the end of the inclusion of this study (March 31, 2023) reporting 864 patients living with acromegaly in Sweden (49% women), whose median (IQR) age was 61 (50-72) years and age at acromegaly diagnosis was 48 (37-58) years. Among those with registered information on biochemical control, 20% were reported to be not biochemically controlled (29). The control group included 139 participants (42 patients and 97 clinical staff) each matched with a specific patient with acromegaly by age (±5 years), gender, and study site. Thus, for 12 patients with acromegaly, a matched control was lacking due to difficulties in recruitment during the study inclusion period. These 12 patients were excluded from digital voice analysis but were included in the description of the acromegaly cohort and the VHI analysis. Baseline information and subgroup analysis in biochemically active and controlled patients with their matched controls is shown in Table 2.

**Table 2.** Baseline characteristics of patients with acromegaly vs controls for all patients, those biochemically active, and those biochemically controlled

Characteristic	All		Biochemically active		Biochemically controlled	
	Acromegaly (n = 151)	Controls (n = 139)	Acromegaly (n = 35)	Controls (n = 32)	Acromegaly (n = 116)	Controls (n = 107)
Age, years	56.9 (44.8-66.7)	55.9 (46.0-66.5)	43.6 (36.0-59.8)	44.3 (35.6-60.3)	58.4 (47.7-67.7)	58.5 (50.2-68.0)
Females	59 (39%)	57 (41%)	15 (43%)	14 (44%)	44 (38%)	43 (40%)
Males	92 (61%)	82 (59%)	20 (57%)	18 (56%)	72 (62%)	64 (60%)
Height, cm	178 (169-187)*	175 (167-182)	176 (169-187)	176 (166-183)	178 (169-187)*	175 (167-182)
Weight, kg	91 (78-105)*	78 (69-91)	90 (78-104)**	77 (70-88)	91 (78-105)***	81 (68-92)
BMI, kg/m^2^	28.0 (25.3-31.2)***	25.1 (23.0-28.6)	28.6 (25.8-31.4)***	25.1 (22.1-27.3)	28.0 (25.1-31.1)***	25.3 (23.1-29.4)
Tertiary education	80 (56%)*	94 (70%)	21 (64%)	23 (77%)	59 (53%)*	71 (68%)

Data presented as n (%) or median (IQR).

Missing data: age (n = 1), height (n = 4), weight (n = 6), and tertiary education (n = 12).

Statistical significance: *P < .05, **P < .01, ***P < .001 vs controls.

Abbreviations: BMI, body mass index; IQR, interquartile range.

### Voice Analysis

**Figure 1.**
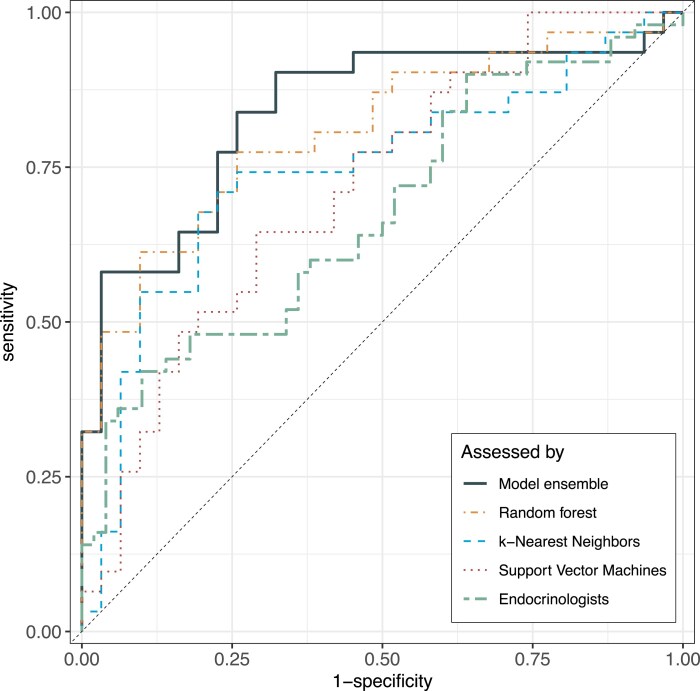
ROC AUCs for model performance. ROC AUCs for the 3 machine learning classification models (random forest, k-nearest neighbors, support vector machine), the stacked model ensemble, and the endocrinologists' assessment. Abbreviation: ROC AUC, area under the receiver operating characteristic curve.

A test set with 31 pairs of participants (ie, 31 patients with acromegaly and 31 matched controls) was randomly selected but intended to have close consistency with the whole study population (ie, 39% [24/62] women and 23% [7/31] with biochemically active acromegaly). In the test set, the model ensemble correctly classified 22 patients with acromegaly and 24 controls but misclassified 9 patients with acromegaly (6 men) and 7 controls (5 men), resulting in a sensitivity of 71%, specificity of 77%, and ROC AUC of 0.84. The ROC AUCs for all models are presented in Fig. 1. Three of the 5 patients in the test set with newly diagnosed acromegaly were misclassified as controls.

### Endocrinologists' Assessment

The 12 endocrinologists' assessment of each participant’s status (50 patients, 50 controls) as acromegaly (Y/N) by listening to 1 sustained [a] and 4 short sentences in random order resulted in a mean sensitivity of 41% and specificity of 75%. The ROC AUC of the machine learning model ensemble was significantly larger than that of the endocrinologists (0.84 vs 0.69, *P* = .0042; Fig. 1). Diagnostic performance by solely listening to text reading was similar (sensitivity 41%, specificity 79%, and ROC AUC 0.70), but poorer by listening only to the sustained [a] (sensitivity 50%, specificity 62%, and ROC AUC 0.57). The sensitivity and specificity of the individual listeners and types of voice stimuli are presented elsewhere (Table S2 (25)). The median intrarater reliability of all listeners was moderate to average with better intrarater reliability in text reading than in sustained [a] (Cohen’s kappa 0.61 vs 0.54), and with a wide variability both for read text (Cohen’s kappa 0.35-0.84) and sustained [a] (Cohen’s kappa 0.00-0.74).

### Voice Handicap Index

**Figure 2.**
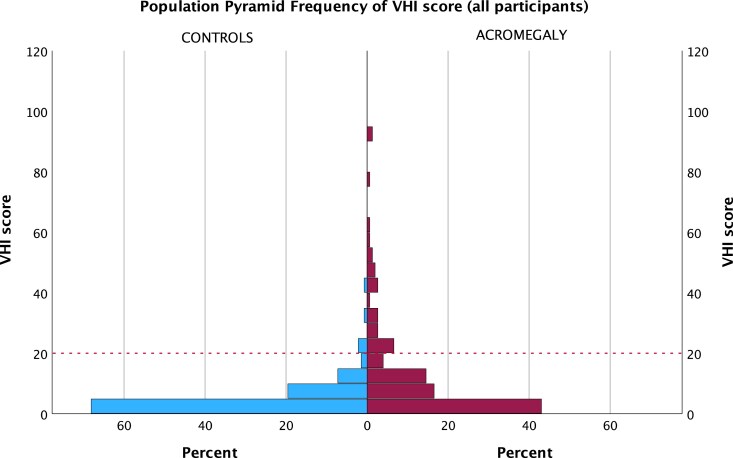
Population pyramid VHI score histograms for the acromegaly and control groups. Each bar represents a 5-point interval for total VHI. The dotted line at VHI 20 indicates the threshold for clinically relevant voice limitations. Statistically significant difference (*p* < .001) between patients with acromegaly and controls. Abbreviation: VHI, Voice Handicap Index.

VHI results were available in all participants except for 1 control. Total VHI score was significantly higher in patients with acromegaly than in the matched controls (median VHI 6 vs 2, *P* < .001; Table 3). Concurrently, the proportion of individuals with VHI ≥20 was significantly higher in the acromegaly group (22.5% vs 3.6%; *P* < .001) with no significant difference between biochemically active and biochemically controlled patients with acromegaly (Table 3). Similar results were observed for all 3 VHI subdomains (ie, significantly larger proportions with a subdomain VHI score ≥10 in the patients with acromegaly than controls), which was most pronounced for the VHI physical subdomain. The frequency distribution of total VHI for the acromegaly and the control groups is shown in Fig. 2. Univariate ordinal regression analyses showed that patients with acromegaly have higher odds for increased VHI score than controls (odds ratio [OR] 2.93, *P* < .001) with similar results in the multivariable model adjusting for age, gender, inclusion site, BMI, and tertiary education (OR 3.16, *P* < .001). Repeated analyses for men and women separately confirmed that these effects of acromegaly were significant in both men and women, with a trend for stronger effect in the women (Table S3 (25)). However, an ordinal regression analysis including an interaction term did not show significant interaction between acromegaly and gender (*P* = .197). In patients with acromegaly, age, time from first symptom to diagnosis, and time in biochemical control were not significantly associated with the total VHI score (ordinal regression).

**Table 3.** Total VHI score and proportions of patients with VHI above thresholds for clinically significant voice limitations for patients with acromegaly vs controls for all patients, those biochemically active, and those biochemically controlled

VHI	All		Biochemically active		Biochemically controlled	
	Acromegaly (n = 151)	Controls (n = 139)	Acromegaly (n = 35)	Controls (n = 32)	Acromegaly (n = 116)	Controls (n = 107)
VHI total	6.0 (1.0-16.0)***	2.0 (0-6.3)	5.0 (0-22.0)	2.5 (0-8.0)	7.0 (1.0-14.6)***	1.0 (0-6.0)
VHI total ≥20	22.5%***	3.6%	28.6%*	6.3%	20.7%***	2.8%
VHI functional ≥10	13.9%***	1.4%	20.0%**	0	12.1%**	1.9%
VHI physical ≥10	22.5%***	3.6%	28.6%*	6.3%	20.7%***	2.8%
VHI emotional ≥10	10.6%**	1.5%	14.3%*	0	9.5%*	1.9%

Data presented as n (%) or median (IQR).

Statistical significance: *P < .05, **P < .01, ***P < .001 vs controls.

Abbreviations: IQR, interquartile range; VHI, Voice Handicap Index.

## Discussion

This is the first study showing that a machine learning algorithm based on a large number of digital voice parameters can identify acromegalic voice changes in a sustained [a] with high accuracy. We also found that patients with acromegaly, both biochemically active and controlled, report more voice symptoms than matched controls. Voice has been investigated as a biomarker for disease detection of cardiovascular, neurological, and mental disorders that may affect voice production or speech regulation (5, 16), but this is the first study evaluating voice as a biomarker for acromegaly detection.

Our data support previous findings of persistent voice changes in active and controlled disease with no significant difference in voice parameters after treatment despite regression of mucosal edema in the vocal tract (7, 10). This indicates that reversible soft tissue alterations are not the only factors affecting the voice in acromegaly. Remaining orodental pathologies, prognathism, and other changes in the viscerocranium after biochemical control of acromegaly may thus contribute to voice and speech changes (15, 30). In our acromegaly cohort, 66% had IGF-1 levels within the reference range, but 77% were assessed as biochemically controlled by an experienced endocrinologist based on full clinical, biochemical, and radiological assessment at the including center. Considering the assay variability in IGF-1 measurements, the compound clinical assessment of biochemically active or controlled was used for subgroup analysis in the manuscript as a more robust indicator of clinical activity (31).

It was difficult for experienced endocrinologists to predict acromegaly by voice recordings of a sustained [a]. When listening to short sentences, endocrinologists showed better accuracy, but still lower than the machine learning model. The variance of accuracy measures between observers and the low intrarater reliability showed a high level of uncertainty in the endocrinologists' prediction of acromegaly by voice. The physician’s prediction may be even less accurate in a real-life setting since physicians primarily seeing patients with undiagnosed acromegaly are usually nonendocrinologists, who are less familiar with acromegaly.

The perceived level of voice impairment in everyday situations caused by acromegaly has not been thoroughly evaluated before. We found that patients with acromegaly report more voice problems than matched controls in all subdomains of the VHI. Although VHI is a consistent tool for assessing the impact of voice disorders on quality of life, previous literature reports poor correlation with objective voice measurements such as acoustic analysis (32). Furthermore, VHI was mainly developed for patients with a voice disorder diagnosis and is not specifically focused on acromegaly-associated voice symptoms, thus leading to low scores for several questions with limited relevance in acromegaly. Taking this into account, we have used conservatively high thresholds (VHI total ≥20, subdomains ≥10) for clinically relevant perceived voice handicap to avoid an overestimation of the effect of acromegaly. However, an underestimation of perceived voice problems in acromegaly cannot be excluded and a more specific questionnaire focusing on acromegaly may be more useful. Taken together, this study indicates that long-term voice problems in acromegaly may need increased attention and interventions by speech and language therapists.

This nationally collected acromegaly cohort represented 17.5% of all known Swedish patients living with acromegaly with similar age, age at diagnosis, and proportion biochemically controlled but slightly lower proportion of women than in the Swedish Pituitary Registry. Considering the high coverage of the Swedish Pituitary Registry, we consider our cohort to be representative of the Swedish acromegaly population (29). The external validity of these findings to other populations needs to be further studied in other ethnicities and languages. The patients in this cohort were studied at different phases of acromegaly from newly diagnosed to a large proportion in long-term biochemical remission, which may impact voice changes. However, acromegaly is a rare disease, and this type of broad acoustic analysis and machine learning requires a large amount of voice data, so that analysis of only biochemically active patients would have limited power. Even with the limitation of few newly diagnosed patients with acromegaly, the model was successfully trained and future studies including more patients with newly diagnosed acromegaly may allow for further training and improvement of the model. Further training of the model with a larger proportion of treatment-naive patients with acromegaly is essential for the development of a diagnostic tool. The use of an easy-access tool in a mobile phone for voice recordings provides an opportunity to easily collect more data to train and evaluate the algorithm to assess voices with suspicion of acromegaly. This may lead to better opportunities for initial acromegaly screening, such as in clinics seeing patients with higher risk for acromegaly such as those with sleep apnea and carpal tunnel syndrome, in public awareness campaigns, and potentially shortening of diagnostic delay (33).

We conclude that high-resolution digital analysis of voice samples collected by a mobile phone and machine learning training resulted in a preliminary algorithm with a high accuracy for acromegaly. This study is the first step to further investigate voice as a biomarker of acromegaly, but more training data and further validation in other populations and languages is essential to create a model with higher sensitivity and specificity.

## Data Availability

Anonymized data is available upon request for the actual except voice recordings due to the sensitive nature of these personal data.

## Abbreviations

BMI: body mass index
IGF-1: insulin-like growth factor 1
IQR: interquartile range
KNN: k-nearest neighbors
N: no
OR: odds ratio
RF: random forest
ROC AUC: area under the receiver operating characteristic curve
SVM: support vector machine
ULN: upper limit of normal
VHI: Voice Handicap Index
Y: yes
